# P-1629. Reduction in *Clostridioides difficile* Over-diagnosis Following Suppression of *C. difficile* Target from Multiplex Molecular Stool Assay in a Pediatric Healthcare System

**DOI:** 10.1093/ofid/ofae631.1795

**Published:** 2025-01-29

**Authors:** Zachary M Most, Kayla Sceeles, Laura Filkins, Mehgan Kidd

**Affiliations:** University of Texas at Southwestern Medical Center, Dallas, Texas; Children's Medical Center Dallas, Dallas, Maryland; University of Texas Southwestern/Children's Health, Dallas, Texas; University of Texas Southwestern Medical Center Dallas, Dallas, Texas

## Abstract

**Background:**

*Clostridioides difficile* testing by polymerase chain reaction (PCR) does not differentiate asymptomatic colonization from infection, and children are frequently colonized with toxigenic strains of *C. difficile*. Over-diagnosis of *C. difficile* infections can lead to adverse patient outcomes and poorer quality metrics for hospitals.

**Figure 1:**
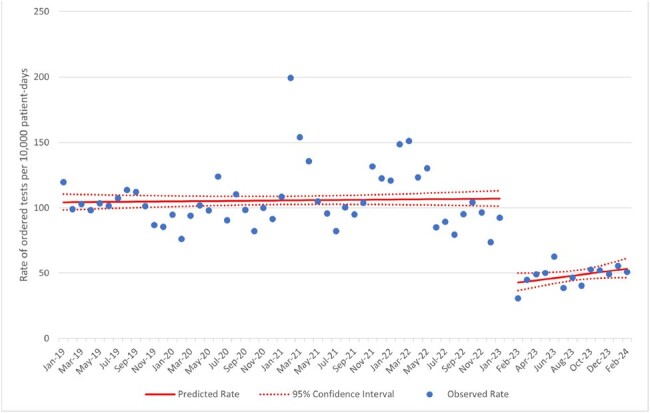
C. difficile PCR orders following change in GI PCR Panel, Interrupted Time Series

**Methods:**

In January 2023 our pediatric health system enacted multimodal changes to the electronic ordering of stool tests for *C. difficile* and other gastrointestinal pathogens. A multiplex PCR test for stool was updated to suppress testing for toxigenic *C. difficile*. The only remaining method to test for *C. difficile* by PCR in house was a *C. difficile* toxin gene specific PCR. Additionally, appropriate clinical criteria with a hard stop were added to the *C. difficile* PCR order (all of: no laxatives in the past 24 hours, at least 3 liquid stools in the past 24 hours, at least one risk factor for *C. difficile* infection, and patient at least one year old). If providers still sought *C. difficile* testing without meeting these criteria, infectious disease specialist approval was required. We analyzed the effect of this intervention using interrupted time series analysis and pre-post aggregate rates from January 2019 through February 2024.

**Figure 2:**
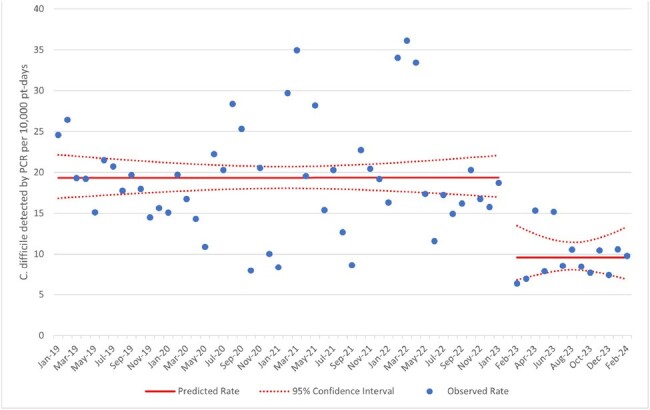
C. difficile positive rate following change in GI PCR Panel, Interrupted Time Series

**Results:**

There were 4426 stool PCR tests performed for *C. difficile* in the pre-intervention period and 622 in the post-intervention period. The mean (± sd) age of those tested was 7.8 years (± 6.1 years). After the intervention, there was an immediate 61% decrease in *C. difficile* testing incidence (95% CI [53% - 67%], Figure 1) and 51% decrease in *C. difficile* diagnosis incidence (95% CI [26% - 67%], Figure 2). There was no significant immediate change or change in slope in the *C. difficile* antibiotic treatment incidence or HFO *C. difficile* incidence, but there was a trend towards lower rates of healthcare facility onset *C. difficile* after the change (aggregate rate 4.13 per 10,000 non-NICU patient-days prior to intervention vs. 3.39 after, *P* = .28).

**Conclusion:**

Removing *C. difficile* from multiplex PCR assays and adding required clinical criteria to the electronic order reduces the number of *C. difficile* tests and diagnoses in a pediatric health system.

**Disclosures:**

**Laura Filkins, PhD**, Avsana Labs: Board Member|Avsana Labs: Stocks/Bonds (Private Company)|Biofire Diagnostics: Grant/Research Support

